# Physiological and morphological effects of a marine heatwave on the seagrass *Cymodocea nodosa*

**DOI:** 10.1038/s41598-022-12102-x

**Published:** 2022-05-13

**Authors:** Alizé Deguette, Isabel Barrote, João Silva

**Affiliations:** 1 Centre of Marine Sciences, University of Algarve, Campus of Gambelas, 8005-139 Faro, Portugal; 2 Faculty of Science and Technology, University of Algarve, Campus of Gambelas, 8005-139 Faro, Portugal

**Keywords:** Climate change, Marine biology, Ecology, Physiology, Plant sciences

## Abstract

Marine heatwaves (MHWs) are increasing in frequency and intensity as part of climate change, yet their impact on seagrass is poorly known. The present work evaluated the physiological and morphological responses of *Cymodocea nodosa* to a MHW. *C. nodosa* shoots were transplanted into a mesocosm facility. To simulate a MHW, water temperature was raised from 20 to 28 °C, kept 7 days at 28 °C, cooled down back to 20 °C and then maintained at 20 °C during an 8-day recovery period. The potentially stressful effects of the simulated heatwave on the photosynthetic performance, antioxidative-stress level and area *vs* dry weight ratio of leaves were investigated. The maximum quantum yield of photosystem II (ΦPSII) increased during the heatwave, allowing the plants to maintain their photosynthetic activity at control level. Negative effects on the photosynthetic performance and leaf biomass of *C. nodosa* were observed during the recovery period. No significant oxidative stress was observed throughout the experiment. Overall, although *C. nodosa* showed a relative tolerance to MHWs compared to other species, its population in Ria Formosa is likely to be negatively affected by the forecasted climate change scenarios.

## Introduction

Heatwaves are often pointed out as yet another negative consequence of climate change and global warming. Only recently, and pressured by the urgency of the climatic threat, the scientific community started qualifying and understanding the marine heatwave (MHW) phenomenon. The Intergovernmental Panel on Climate Change (IPCC) defined MHW as “an event at a particular place and time of the year that is rare and predominately, but not exclusively, defined by a relative threshold; that is, an event rarer than 90th or 99th percentile of a probability density function”, in the Special Report on the Ocean and Cryosphere (SROC)^1^. MHW occur at any time of the year, everywhere in the world, for a period ranging from a few days to several weeks at varying intensities. Their frequency and intensity have severely increased in the past century^2^, causing massive extinctions events (e.g. “The Blob”, a large mass of high-temperature water in the Pacific Ocean that killed nearly a million seabirds in Alaska and California in 2015–2016)^3^, range shifts^4^ and coral bleaching (mass coral mortality occurred in the Great Barrier Reef in 2016, with losses exceeding 50%)^5–8^.

Seagrasses are angiosperms (flowering plants) adapted to marine life, accounting for about 60 species worldwide^9^. These clonal plants, which shoots grow from the expansion of rhizomes^10^ and are found worldwide along tropical, temperate, and boreal latitudes, except in the Antarctic region, colonising intertidal areas and shallow waters in subtidal zones^11^. Seagrass meadows are among the most ecologically valuable estuarine and coastal ecosystems, providing a large range of essential ecosystem services. They offer valuable feeding, spawning and nursery grounds for numerous species of flora and fauna^12^ and enhance the production and biodiversity of adjacent ecosystems^13^. Seagrass beds slow down the currents, trap suspended particles and, in this way, increase light penetration^14^. They have been increasingly studied because of their nitrogen fixation capacity^15^, their role as carbon sinks^16–19^, and their major importance in “blue carbon” sequestration and export^20^. Although they represent less than 0.1% of the ocean surface, seagrasses are responsible for 20% of oceanic carbon sequestration globally^21,22^. Regrettably, seagrass meadows are declining globally at a dramatic rate (110 km^2^ year^−1^ since 1980 i.e., 5% year^−1^ globally or at least 1/3 since World-War II)^23,24^ because of the environmental changes they face. Notably, MHWs have been proved to be one of the major drivers of seagrass decline (36% of Shark Bay’s—Western Australia—seagrass meadows were negatively affected by a MHW in 2010/11)^25^. In fact, a temperature increase above the optimum temperature of seagrasses reduces the photosynthetic rate and leave’s productivity and increases respiration, photosynthetic stress responses and biomass losses^26,27^. In addition, these effects are even more considerable as the number of thermal stress days increases, with species-specific responses^27,28^. Previous studies showed that heat stress negatively impacts the metabolism of *Zostera noltii*^29^ and *Zostera marina*^30,31^, lowering the flowering and reproductive intensity of this species^32^ and leading to important shifts in physiological stress indicators (inhibition of photosynthetic efficiency (-23.9%), increased respiration (+ 58.3%) and decreased carbohydrate decomposition products)^33^. A study showed that the species *Cymodocea nodosa* and *Posidonia oceanica* were able to recover from a simulated MHW, with shifts in carbon allocation strategies that differ between shoots coming from different thermal environments (thermal adaptation)^34^. *C. nodosa* showed the ability to change its metabolism to handle short thermal stress (shifts in photosynthetic pigments concentration, increased antioxidant activity and CO_2_ assimilation)^35^.

In Europe, 35 684 ha of seagrass meadows disappeared between 1869 and 2016, i.e., 29% of the documented area^36^. The same study showed that the highest proportions of declines were reported for the species *Z. marina* and *C. nodosa* (net losses of 57% and 46% of the documented area, respectively). Despite the general decline of seagrasses in Europe between 1869 and 2016, gain was reported in the 2000s, for the first time since the 1950s (20% net gain in area per decade in the 2000s)^36^, showing a significant trend reversal as a result of conservation and restoration actions. Thus, seagrass decline is neither irreversible nor to be generalised: hope remains to maintain or even improve the services they provide.

*C. nodosa* (Ucria) is the most abundant subtidal seagrass species in Ria Formosa coastal lagoon, Southern Portugal, where it covered an area of 0.913 km^2^ in 2007^37^. It is a temperate-warm adapted species, resistant to relatively high-water temperatures (25–32 °C)^38,39^. Its distribution ranges from its northern limit on the southern Portuguese coast to its southern limit in Senegal^40^. As a tropical-originated species, *C. nodosa* has a high optimum temperature range (24.5 and 31.0 ± 0.5 °C for growth and photosynthesis, respectively) when compared to temperate species in the Mediterranean, such as *Z. marina* (15.3 ± 1.6 and 23.3 ± 1.8 °C, respectively)^41^. While the effects of MHWs and heat shocks have been studied mainly on *Z. marina*^31,33,42,43^, they have been poorly studied for *C. nodosa*. Based on the existing literature, we hypothesize that negative effects of MHWs are also to be expected on this species (e.g., lower productivity/photosynthetic activity, increased oxidative damage), despite its potential ability to thrive in warm waters.

The objective of this work was to investigate the morphological changes and physiological capacity of *C. nodosa* to cope with MHWs in Ria Formosa, in the framework of evaluating and forecasting the species tolerance under climate change scenarios that include more frequent and intense MHWs. Specifically, we investigated the plant’s physiological and morphological response throughout the peak of a MHW and the existence of putative sequels, evaluated during a recovery period. The absolute temperature value used for the MHW simulation was based on in situ temperature records and the definition and classification of MHWs. The photosynthetic performance of different tissue ages, the antioxidant activity, the potential oxidative damage and changes in leaf biomass were assessed.

## Results

### Photosynthetic activity

#### Photosynthesis-Irradiance (P-I) curves

**Figure 1:**
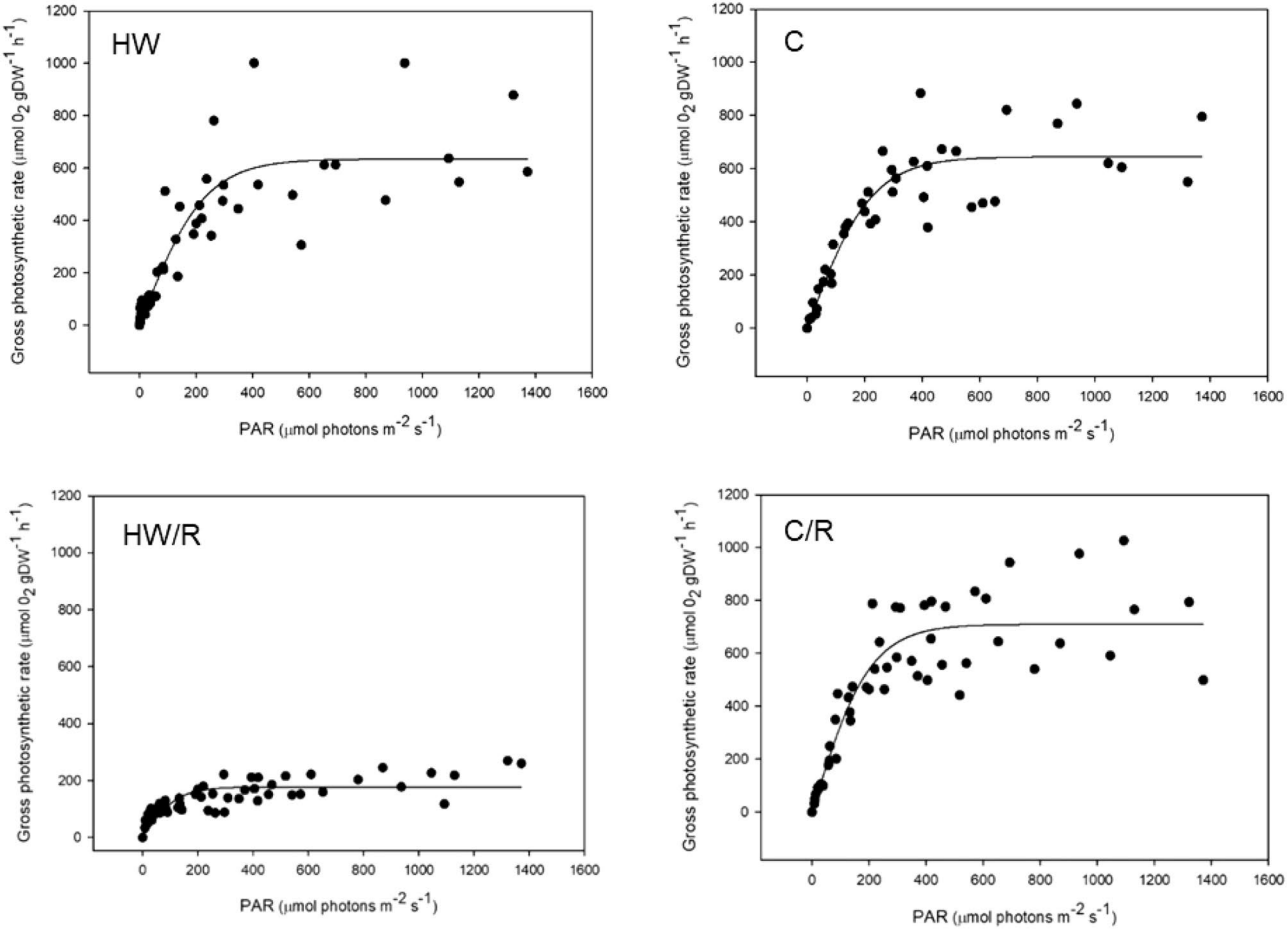
P-I curves of *C. nodosa*’s leaves. Leaves were sampled during the heatwave peak (heatwave, HW, and control, C) and after a 7-day recovery (HW/R and C/R). Data were fitted with the Jassby & Platt (1976) model equation.

*C. nodosa* leaves’ photosynthetic activity responded to light stimulation by a typical Photosynthesis-Irradiance (P-I) hyperbolic-shaped response with increasing light intensity (Fig. 1).

The photosynthetic parameters α, P_m_ and I_k_ were significantly lower in leaves from plants recovering from the heatwave than control (*p* < 0.001, *p* < 0.001 and p = 0.008, respectively; Table 1).

**Table 1:** *C. nodosa’s* photosynthetic parameters obtained after fitting the data with the Jassby & Platt (1976) P-I model.

Treatment	Parameters				
	α ± SE	P_m_ ± SE	I_k_ ± SE	R^2^	*n*
**Heatwave**					
HW	2.805 ± 0.352	635.02 ± 37.05	226.4 ± 31, 3	0.809	55
C	2.921 ± 0.305	644.9 ± 27.5	220.8 ± 24.9	0.856	44
Significance level	n.s	n.s	n.s		
**Heatwave recovery**					
HW	1.429 ± 0.229	177.4 ± 8.1	124.2 ± 20.7	0.661	54
C	3.539 ± 0.372	709.8 ± 27.6	200.6 ± 22.5	0.841	55
Significance level	***	***	*		

Mean photosynthetic quantum efficiency (α; μmol O_2_ gDW^−1^ h^−1^/μmol photons m^−2^ s^−1^), maximal photosynthetic rate (P_m_; μmol O_2_ gDW^−1^ h^−1^), and half-saturation irradiance (I_k_; μmol photons m^−2^ s^−1^) are expressed as values ± SE, for each treatment, with corresponding R^2^ and number of observations (*n*). The significance level is the degree of significant difference between treatments HW and C (n.s.: non-significant; *: significant, *p* < 0.05; ***: highly significant, *p* < 0.001), both during the heatwave and after heatwave recovery.

While α was *ca*. twofold lower than control, P_m_ was *ca.* fourfold times lower and therefore, I_k_ decreased significantly. Conversely, no significant difference was observed in leaves sampled during the heatwave.

#### Chlorophyll fluorescence imaging (CFI)

**Figure 2:**
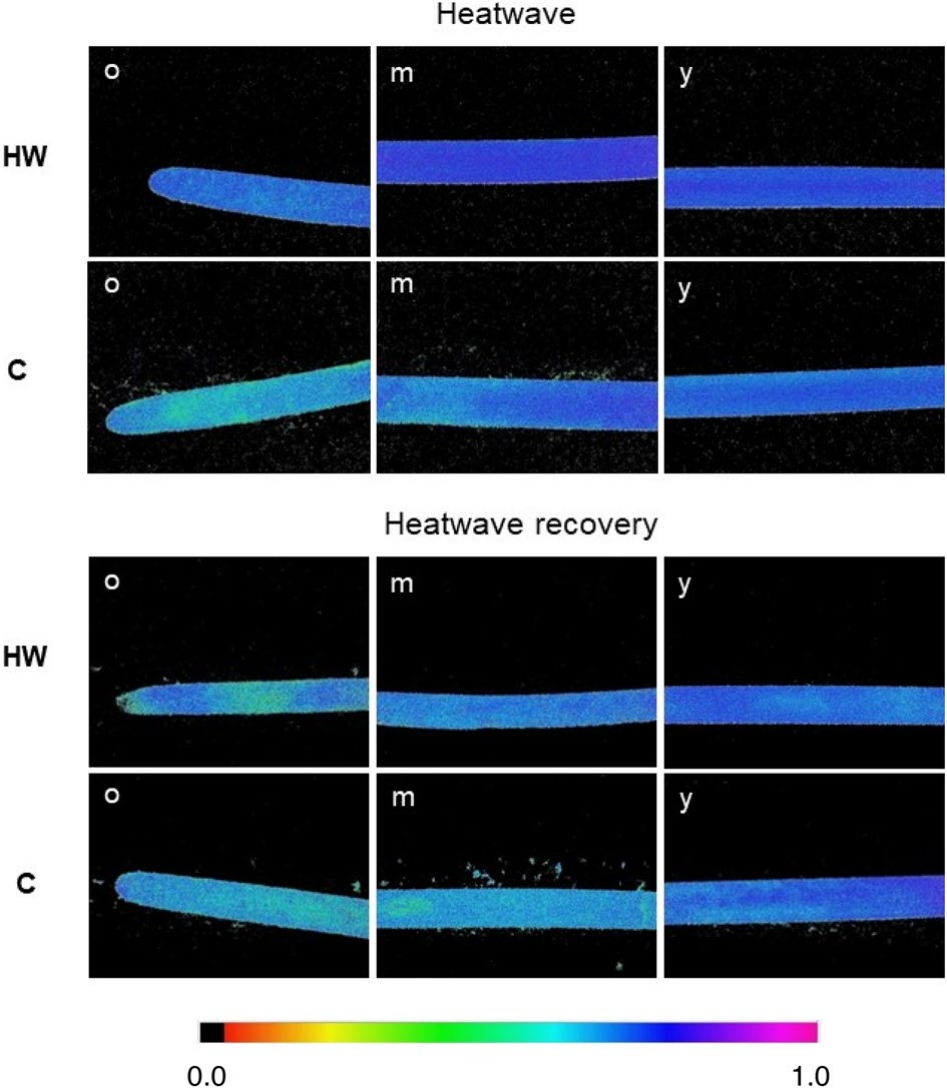
Examples of CFI pictures of *C. nodosa*’s Φ_PSII_, after a saturating light pulse. CFI was done on mature leaves sampled from shoots cultivated in heatwave (HW) and control (C) conditions, both during the heatwave and after heatwave recovery. o, m and y indicate different leaf parts (old, mature and young, respectively). Colour bar on the bottom indicates Φ_PSII_ values ranging from 0.0 (black) to 1.0. (pink). Pictures are 24 × 32 mm (6 × magnification).

The Chlorophyll Fluorescence Imaging (CFI) pictures allow to measure, visualize and pinpoint potential differences in the effective quantum yield of electron transport through photosystem II (Φ_PSII_), along the leaves’ surface and between different tissue ages (Fig. 2).

**Figure 3:**
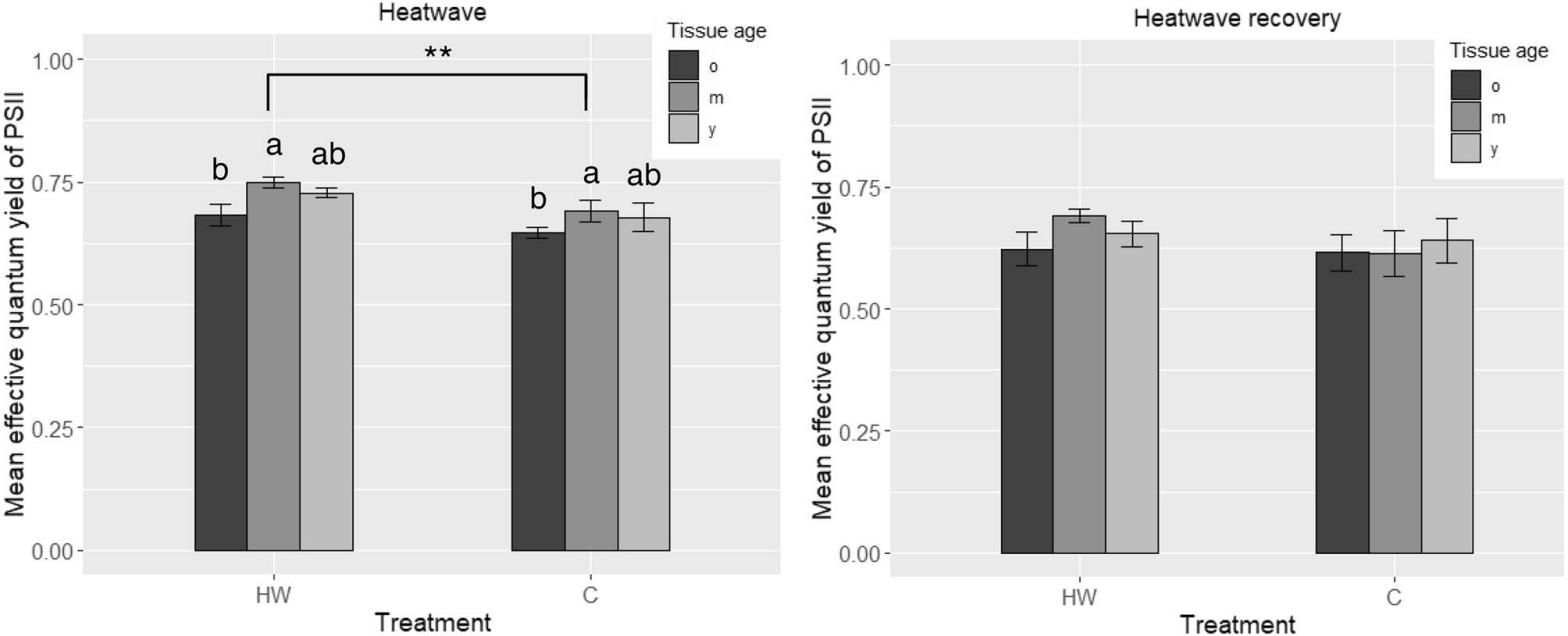
Effective quantume yield of PSII (Φ_PSII_) of *C. nodosa*’s leaf tissues. Old (o), mature (m) and young (y) leaf tissues were sampled from shoots cultivated in heatwave (HW) and control (C) conditions, both during the heatwave and after heatwave recovery. Values are mean ± SE (*n* = *5*). Different letters indicate significant differences between tissue ages (*p* < 0.05), and ** indicates significant differences between treatments HW and C (*p* < 0.01).

During the heatwave, Φ_PSII_ was significantly higher in heatwave (HW) leaves than control (C) (p = 0.008) and old leaf tissues displayed a lower Φ_PSII_ than mature ones in both HW and C leaves (p = 0.035; Fig. 3). No significant differences were found between HW and C leaves nor between tissue ages during the heatwave recovery.

#### Oxidative stress

There was no significant variability in the concentration of oxidative stress indicators in *C. nodosa*’s leaf tissues between treatments HW and C (Table 2).

**Table 2:** Total phenols (mg gDW^−1^), TEAC (μmol Trolox eq gDW^−1^), ORAC (μmol Trolox eq gDW^−1^) and MDA (nmol gDW^−1^) concentrations in mature *C. nodosa*’s leaves from heatwave (HW) and control (C) tanks, during the heatwave and after recovery.

	Treatment			
	*Heatwave*		*Heatwave recovery*	
	HW	C	HW	C
Total phenols (mg gDW^−1^)	11.81 ± 1.72	10.93 ± 0.23	10.79 ± 0.92	10.17 ± 1.41
	*n* = *5*	*n* = *4*	*n* = *5*	*n* = *5*
TEAC (μmol Trolox eq gDW^−1^)	9.47 ± 3.48	9.95 ± 3.09	12.22 ± 1.36	7.96 ± 1.83
	*n* = *5*	*n* = *5*	*n* = *5*	*n* = *5*
ORAC (μmol Trolox eq gDW^−1^)	139.24 ± 34.32	206.01 ± 62.66	132.27 ± 51.08	126.98 ± 13.46
	*n* = *5*	*n* = *5*	*n* = *5*	*n* = *5*
MDA (nmol gDW^−1^)	88.71 ± 20.81	83.49 ± 20.62	186.58 ± 2.14	100.85 ± 34.09
	*n* = *4*	*n* = *4*	*n* = *3*	*n* = *5*

Values are means ± SE and *n* is the number of replicates.

Although non-significant, total phenols and MDA concentration were slightly higher in HW leaves than C leaves during the heatwave, whereas TEAC and ORAC concentrations were slightly lower in HW leaves than control. On the other hand, total phenols, TEAC, ORAC, and MDA concentrations were slightly higher in HW leaves than control during the heatwave recovery.

### Leaf area vs dry weight ratio

**Figure 4:**
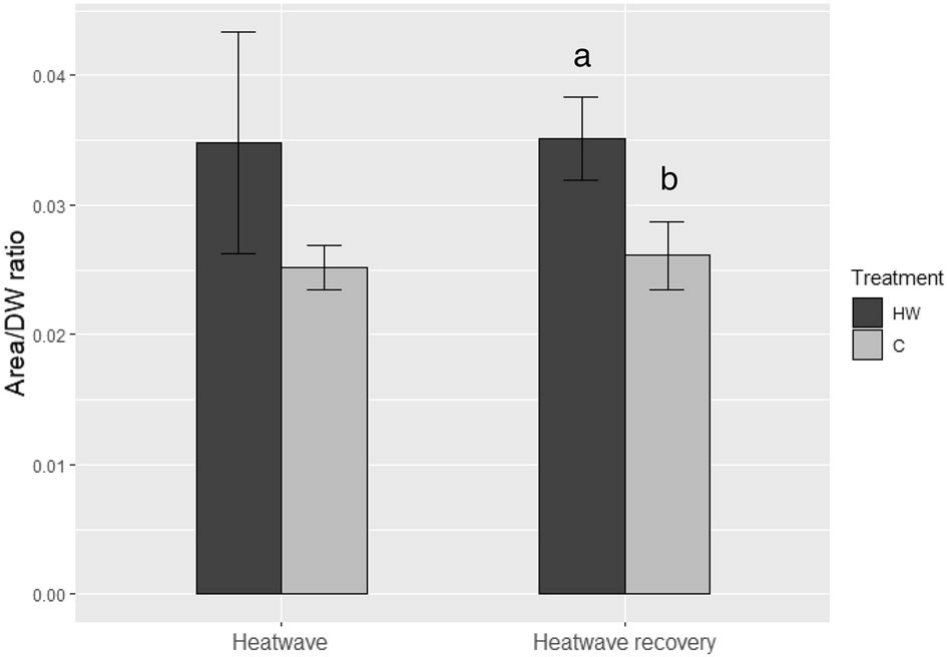
Area *vs* dry weight (DW) ratio of *C. nodosa*’s leaf tissues. Leaf tissues from shoots cultivated in heatwave (HW) and control (C) conditions were sampled both during the heatwave and during heatwave recovery. Values are means ± SD (*n* = *5*). Different letters indicate significant differences (*p* < 0.01).

*C. nodosa*’s leaf area *vs* DW ratio was significantly higher in HW than C leaf tissues during the heatwave recovery (Fig. 4). Hence, for the same leaf area, *C. nodosa*’s leaves that went through a heatwave simulation had less biomass than those grown in control conditions.

## Discussion

Our results show that MHWs (in this case, a seven-day spring heatwave peaking at 28 °C) have a negative impact on *C. nodosa*’s physiology, but the effects may only become evident in the aftermath of the heatwave peak. Reduction of the photosynthetic capacity and light-saturating irradiance was observed 7 days after the end of the heat stress, along with a decrease in leaf biomass. Coupled to the reduced photosynthetic rates and efficiency, this leaf biomass loss implies an additional reduction of the global productivity of the plants, with direct consequences on growth. On the other hand, the heatwave did not imply significant oxidative damage or changes in the leaves’ antioxidant system of *C. nodosa*.

While the heatwave peak only affected Φ_PSII_ significantly, the measured photosynthetic parameters showed a strong decrease after 7 days of recovery at lower temperature: the maximal photosynthetic rate (P_m_), the amount of O_2_ released per unit of incident light (α) and the minimum-light intensity needed to reach P_m_ (I_k_) dropped significantly during the recovery period. Conversely, no changes in these photosynthetic parameters were observed during the heatwave, showing that the effects only became apparent several days after the alleviation of the heat stress. Φ_PSII_ increased significantly during the heatwave, accounting for a higher electron transport rate in the electron transport chain of photosynthesis^44^ under the heat stress provoked by the MHW. Maintaining a high electron flow in the electron transport chain is advantageous to compensate the negative effects of heat stress and keep photosynthetic and growth rates at a constant level. Φ_PSII_ was higher in mature parts of the leaves than in old tissues, which has been shown to be a common feature related to the reduction of leaf thickness and cell layers towards the tip^45^. Nonetheless, in our work, this feature was not noticeable during heatwave recovery, which may be related to the increased area *vs* DW ratio in heatwave recovering leaves, as discussed below. When Φ_PSII_ returned to control level several days after heatwave relief, the photosynthetic performance of the plant dropped, suggesting that *C. nodosa* is only able to temporarily maintain its gross photosynthetic activity at a normal rate under thermal stress as a “compensation” response, by increasing the electron transport rate through PSII during a few days. However, it is likely to be unable to sustain this metabolic compensation response in the long term, resulting in the drop of photosynthetic performance several days after the heat stress, while Φ_PSII_ drops back to control level. Costa et al. also suggested that Φ_PSII_ increases with heat stress in *C. nodosa* leaves (after a 4-day heat shock at 40 °C)^35^. This study confirms that a short-term response to heat stress involves an increase in Φ_PSII_, probably to support photosynthesis during thermal stress. The fact that Φ_PSII_ returns to control levels after the heatwave suggests that *C. nodosa*’s PSII has a certain ability to recover from the heat-stress damage^39^. However, nothing suggests that it would be able to recover from a more intense and long-lasting MHW, as those forecasted in future climate-change scenarios. Costa et al. also suggested that P_m_ was lower in *C. nodosa* shoots that had suffered from intense thermal stress (4 days at 40 °C) than in plants kept at 20 °C^35^. While both a 4-day heat shock at 40 °C and our 7-day heatwave at 28 °C had negative consequences on the photosynthetic activity of *C. nodosa* in Ria Formosa, the responses seem to appear at different time scales (right after the heat shock and after a 7-day recovery, respectively). We suggest that a prolonged, but less intense temperature rise (namely the MHW), may have delayed consequences on the plant’s photosynthetic activity, whereas a short, although more intense heat stress, involves an immediate decrease of P_m_, and with it, the immediate drop of photosynthetic efficiency. Yet, the resilience of *C. nodosa* to MHWs must be investigated on a longer time scale (e.g., after a more extended recovery period) to know whether this species can entirely recover from the heatwave or if such consequences are irreversible.

The unchanged photosynthetic parameters, together with the increase in Φ_PSII_ in leaves during the heatwave, may be related with the increase of O_2_-independent electron flow, such as the cyclic electron flow within PSII, or the water-water cycle (PSI) that does not imply net O_2_ uptake while regenerating the ascorbate needed for antioxidant protection^46^ and allowing extra ATP synthesis. The decrease of photosynthetic performance in heatwave-recovering leaves suggests that a higher fraction of the oxygen produced by photosynthesis is consumed during the heat stress recovery, indicating the up-regulation of the oxygen-consuming process(es), such as respiration and photorespiration^47,48^. The increment of these oxygen-consuming processes can in turn enhance the production of reactive oxygen species (ROS) such as H_2_O_2_ and superoxide radicals^49,50^. Although it has been reported that thermal stress induces the production of ROS in submerged macrophytes^51^, the putative consequences of increased oxidative stress were not observed here, as neither membrane lipid peroxidation (measured as MDA) nor the ROS scavenging capacity (TEAC and ORAC) presented significant changes during and after the heatwave. Similarly, the unchanged MDA values show that the possible increase in ROS did not provoke oxidative stress, meaning that the existing antioxidant system of *C. nodosa* was probably sufficient to avoid oxidative damage. Yet, we cannot entirely rule out the possibility that stress was not detected due to putative limitations of the parameters analysed.

Although non-significantly, total phenols, TEAC, ORAC and MDA concentrations were slightly higher in leaves recovering from the MHW than in control ones. While the short-term effects of the heatwave on biochemical oxidative-stress indicators were not clearly shown in this experiment, the consequences appear to be more relevant during the recovery. This could be interpreted as a long-term acclimation response of *C. nodosa*’s biochemistry to the potentially low oxidative stress caused by the prolonged heat stress. Costa et al. suggested that a short and intense heat stress (40 °C for 4 days) implies a significant increase in *C. nodosa*’s antioxidant response (measured with TEAC)^35^. The non-significance of our results may be justified by the characteristics of the MHW or heat shock, which, in the same study, was more intense and suddenly imposed, whereas in the present work, the MHW was progressively imposed and less intense. The absence of oxidative damage (measured with MDA) under a short and intense heat shock^35^ is in line with our results. Indeed, MDA concentration did not increase in *C. nodosa* leaves exposed to thermal stress, suggesting that *C. nodosa* is resistant to the stress caused by MHWs of different lengths and intensities. Nonetheless, foliar MDA is slightly higher after recovery from a prolonged heatwave (ca. 190 nmol gDW^-1^; present study) than right after a short and intense heat shock (ca. 100 nmol gDW^-1^^35^). These results suggest that oxidative damage is likely to be more important in plants recovering from heatwave-type stress (more extended heat stress, long-term effect) than immediately after a short heat shock. In Ria Formosa, *C. nodosa* seems to have a sufficient antioxidant capacity to cope with stress induced by a spring MHW. Yet, a longer and/or more severe MHW for this time of the year may eventually significantly increase the oxidative stress and cell damage in *C. nodosa* leaves.

This study showed that the simulated MHW also impacted the morphology of *C. nodosa*’s leaves, by inducing a decrease in leaf biomass, especially several days after the end of the heatwave, as shown by changes in the area *vs* DW ratio. *C. nodosa* might have responded to the thermal stress by a reduction in its leave’s thickness, which could be explained by a lower photosynthetic performance and, therefore, a lower growth rate. Another possibility is the increase of the size of the aerenchyma. Aerenchyma lacunae act as either sources or sinks for O_2_^52,53^, and an increase in their volume could be related to a higher O_2_ transfer inside the plant, (from leaves to roots and rhizomes) together with enhanced electron transport (as seen before) or stand for more intense gas exchange for respiration/photorespiration processes. Cross-section analysis of *C. nodosa*’s leaves^45^ would be helpful to further investigate the effect of MHWs on the leave’s anatomy and, more precisely, observe changes in the size of the aerenchyma^54,55^.

The present study investigated the effects of a particular spring-like MHW in Ria Formosa on *C. nodosa* shoots. Results must not be extrapolated for all-year-round conditions or for shoots coming from different thermal environments (temperate or tropical/subtropical). In fact, the optimum temperature for seagrass growth and photosynthesis does not only varies between species but also between individuals of the same species coming from different origins^41^, and metabolic responses of the plants to MHWs can greatly vary with their historic thermal environment^31,34,56,57^. Also, seagrasses may have different responses to MHWs, whenever these events occur at different times of the year (summer/winter), as the plant’s metabolism follows a seasonal pattern^58^. In the case of reoccurrence of MHW events in a relatively short time, *C. nodosa* and other seagrass species are likely to be more susceptible and more critically affected by heat stress. In fact, Saha et al. showed that cumulative heatwave events have a negative impact on the growth and leaf production rate (*i.e.*, biomass) of *Z. marina,* whereas an isolated MHW event did not induce any significant change in the plant’s biology^59^.

Overall, studies show that *C. nodosa* seems to present a higher tolerance to anomalous temperature events than other seagrass species in the same thermal environment^28,38,56,60^. Although *C. nodosa*’s optimal temperatures are higher than other seagrass species, a spring-like heatwave such as the one simulated in the present work have the potential to negatively impact *C. nodosa* population in Ria Formosa if occurring during a period when seasonal temperatures are lower (e.g., in autumn or early spring). Investigate MHWs’ effects at different times of the year is thus needed to test this hypothesis. In the complex nature realm, an array of biotic and abiotic parameters interacts with the potential to induce synergistic or antagonistic effects^61,62^. Hence, analysing the effects of one parameter (here, the temperature) does not necessarily allow forecasting one species’ response in its natural environment, and conclusions must be withdrawn with caution. Although temperature is the most important factor affecting its production, *C. nodosa* expresses a large variety of responses to different combinations of factors^63^. Therefore, multifactorial experiments are needed to predict more accurately the responses of seagrasses to environmental stressors, like temperature. Finally, comparisons with previous studies must be taken carefully because of the lack of homogenization in methodologies and experimental designs.

## Methods

### Study site

The Ria Formosa coastal lagoon, located in the south coast of Portugal (36°58’N, 8°02’W to 37°03’N, 7°32’W), is an 84 km^2^ shallow mesotidal lagoon. It has been a Natural Park since 1987 and is a Ramsar and Natura 2000 protected area. It is one of the most important wetlands in Europe, spanning over 55 km long and a maximum of 6 km width^64^. The lagoon is 2 m deep on average, and its tidal amplitude varies between 3.50 m on spring tides and 1.30 m on neap tides^65^. It is separated from the Atlantic Ocean by five dynamic barrier islands and two peninsulas and is linked to it by seven channels, five natural and two artificial, allowing water exchange with the ocean. Essentially composed of salt marshes and mudflats in the intertidal and shallow channels in the subtidal, the highly productive Ria Formosa hosts a rich diversity of fauna and flora. It is an important nursery hotspot and feeding ground for many fish and mollusc species^66^, which gives the lagoon high ecological importance. Mean air temperature is 25 °C in summer and 12 °C in winter, which gives Ria Formosa a Mediterranean climate, despite being situated on the Atlantic coast^64^. In this mesotidal system, May–June seawater temperature commonly ranges between 18 °C and 30 °C (https://www.hidrografico.pt/boias). However, in intertidal pools and shallow subtidal areas, the thin water column (especially during low spring tides), coupled to high air temperature and high irradiance, drives the water temperature to rise dramatically, especially in summer^29^ when it can reach 35 °C (João Silva, personal communication). Such drastic environmental changes can significantly affect the physiology and survival of most species found in the Ria Formosa, including seagrasses.

### Plant collection

*C. nodosa* plants, including rhizomes, roots, apical meristem, and shoots with 3–4 leaves each, were carefully collected in Ria Formosa’s Ramalhete channel on May 3rd, 2021 (Supplementary Fig. S1). Following collection, shoots were kept in seawater in closed dark tanks until transplantation into a mesocosm facility within 24 h of uprooting. The collection and use of seagrasses and other plants for experimental purposes in Ria Formosa Natural Park is regulated by ICNF (Institute for Nature Conservation). Therefore, in order to comply with national legislation, a permit (Licença nº17/2021/Recolha) was issued for the collection of all the plant material used in this study. All methods were carried out in accordance with relevant national and international guidelines. A voucher specimen was deposited in the Herbarium of Universidade do Algarve (ALGU) with accession number 15779.

### Experimental setup

An indoor mesocosm experiment was conducted in the Ramalhete station. Ten 65-L plastic tanks (5 replicates per treatment, *n* = 5) were filled with 15 cm of sand collected from Faro beach and supplied with water pumped from Ria Formosa through an open circuit (Supplementary Fig. S2). To reduce microalgae development and contamination in the facility, water pumped from Ria Formosa flowed through a 50-W UV filter before entering the circuit. The water temperature in the circuit was controlled with a temperature controller (ECLI20MA IKOMFORTRC900 inverter, i-Komfort, Kripsol, Toledo, Spain). It flowed into the aquaria at 14 L h^-1^ and was entirely renewed every 5 h. The aquaria were aerated with a bubbling air pipe, and water was kept in motion and homogenised with a water fan. The light above each tank was provided by LED lamps (Ledvance Flood LED 50 W/6500 K WT, Augsburg, Germany) hung above each tank in such a way as to provide approximately the same light intensity in the spectral range from 400 to 700 nm to each aquarium. Light intensity in this spectral range was measured and calibrated before starting the experiment with an LI-250A Light Meter and an LI-190R sensor (LiCor, USA) and ranged from 101.6 to 130.8 µmol m^2^ s^−1^ (113.2 µmol m^2^ s^−1^ on average) just on top of the water surface. To simulate the natural conditions, the lights were automatically turned on at 6 a.m. and off at 9 p.m. (light: dark photoperiod of 15 h: 9 h). The day following harvesting, *C. nodosa* shoots were carefully cleaned from epiphytes and 25 shoots were placed in each aquarium under controlled light and temperature conditions.

**Figure 5:**
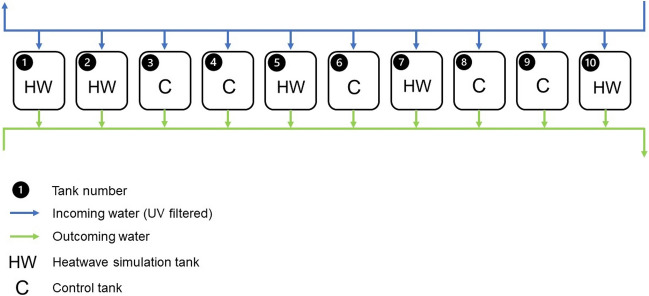
Schematic representation of the experimental setup. Tank number, random treatment assignation (Control, C and Heatwave, HW) and water flow direction (open circuit).

Two treatments (control, C and heatwave, HW) were randomly assigned to the aquaria (Fig. 5).

After the transplant, shoots were left 33 days at 20 °C (1 °C above the water temperature during collection) to allow the plants to acclimate to their new environment. While the C aquaria were kept at 20 °C during the experiment, the MHW simulation was applied in the HW aquaria. The temperature was daily monitored throughout the experiment in all tanks, at the warmest time of the day (between 2 p.m. and 4 p.m.), with a manual thermometer. The temperature was monitored and controlled during the heatwave simulation in each of the five HW tanks with a temperature controller (AquaMedic T controller twin, Bissendorf, Germany).

### Heatwave characteristics

Determination of the maximum temperature (T_max_) of the simulated MHW was based on previous in situ temperature records and the definition of MHWs given by Hobday et al.^67,68^. Temperature data previously recorded in *Caulerpa prolifera* beds (mean daily temperature data, from 10–06-2017 to 17–06-2019; de los Santos, personal communication) was used to get a proxy of the yearly temperature in *C. nodosa* beds in Ria Formosa, as both species are found at a similar depth^69^.

To assess the oceanic SST and the occurrence of oceanic MHWs in Ria Formosa, the NOAA Optimum Interpolation Sea Surface Temperature (OISST) dataset (Huang et al.; methodology developed by Reynolds et al. and described by Banzon et al.)^70–72^ was used. The daily temperature dataset, displayed on the Marine Heatwaves Tracker app^73^, contains the oceanic sea surface temperature (SST), climatology, threshold^67^ data and the MHW events record from 1982 to the present day. A pixel close to Ria Formosa’s inlet channel (Lon = 7.875°W, Lat = 36.875°N) was selected, and a time series was plotted for the oceanic SST from 10–06-2017 to 17–06-2019 (Supplementary Fig. S3).

To determine the temperature inside Ria Formosa during a heatwave event and the potential difference to oceanic MHWs, the correlation between the temperature inside and outside Ria Formosa was established for the year 2018 (R^2^ = 0.885; Supplementary Fig. S4), and the SST climatology inside Ria Formosa was then extrapolated (Supplementary Fig. S5). The occurrence of oceanic MHWs close to Ria Formosa for this time of the year was prospected in the historical data available at the Marine Heatwaves Tracker website (Supplementary Fig. S6). At his location, MHWs happen at any time of the year and have been intensifying in the last decade. MHW events in the Ria Formosa area in the past years during the April-June period were of Moderate intensity (category I; MHWs classification by Hobday et al.)^68^. Nonetheless, there is a global trend toward the increasing frequency of Strong intensity (category II) MHWs^68^. Moreover, the temperature in Ria Formosa’s shallow areas can increase dramatically (João Silva, personal communication) until locally reaching the temperature corresponding to MHWs of Severe and Extreme intensity (category III and IV). An event of Extreme intensity was chosen in this experiment to simulate the dramatically high temperatures of shallow waters and observe its impacts on the seagrass’ metabolism, as a simulation of what is likely to happen in the future according to the MHWs prediction scenarios^74^.

Following the heatwave characterisation and classification proposed by Hobday et al.^68^, an MHW of Extreme intensity (category IV) is characterised by a peak temperature reaching at least 4 × the 90th percentile difference from the mean regional climatology value. We applied this principle to the extrapolated Ria Formosa’s temperature dataset (Supplementary Fig. S7). Between April 1st and June 30th, an MHW of Extreme intensity in Ria Formosa peaks at least at 25.9 °C. However, as said before, water temperature can increase dramatically above this value in some shallow areas of Ria Formosa, such as the smaller channels. Hence, choosing 28 °C as the peak temperature is relevant to simulate an Extreme-intensity MHW in Ria Formosa’s shallow water conditions.

**Figure 6:**
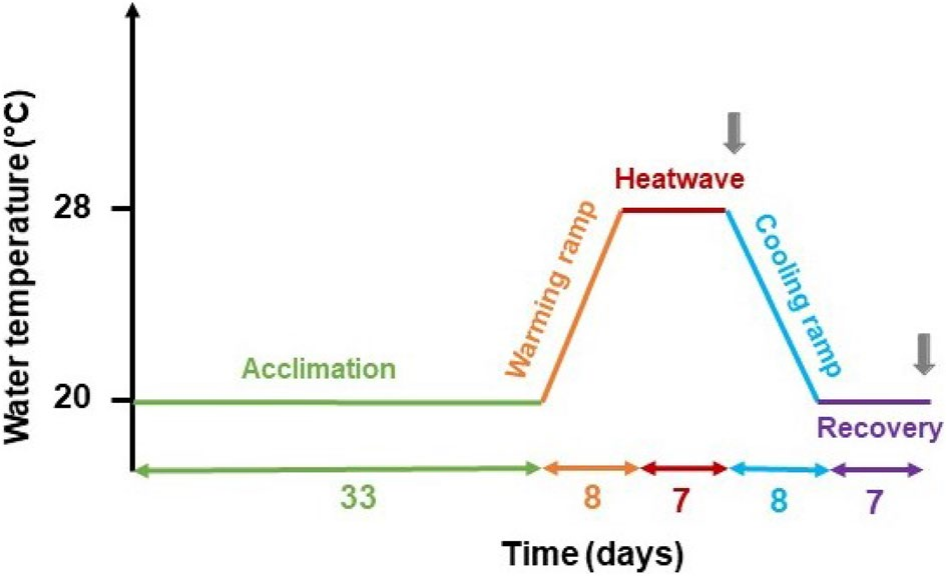
Scheme of the experimental schedule. Water temperature as a function of time. *Acclimation*, *warming ramp*, *heatwave*, *cooling ramp*, *recovery,* and respective duration on the x-axis (in days). Grey arrows indicate the two sampling times.

According to the definition given by Hobday et al.^67^ a MHW has a duration of at least 5 days. Hence, the MHW simulated in this experiment was designed with a seven-day duration and a peak temperature of 28 °C to simulate a spring-like MHW event of Extreme intensity in Ria Formosa’s shallow channels. The experiment’s timeline is described in Fig. 6. After the acclimation period, water temperature was increased from 20 °C to 28 °C, by 1 °C a day during eight days (“*warming ramp*”), maintained at 28 °C for seven days (“*heatwave*”), and then decreased back to 20 °C by 1 °C a day (“*cooling ramp*”). Then, plants were allowed to recover from the heatwave for seven days at 20 °C (“*heatwave recovery*”).

### Sampling design

Samples were collected from each tank (HW, *n* = 5 and C, *n* = 5) at the end of the heatwave peak (“*heatwave”*) and at the end of the recovery period (“*recovery*”; Fig. 6).

Whole mature leaves (the 2nd or 3rd youngest leaf from each shoot) were collected for CFI analysis. The middle part of mature leaves was collected for P-I curves, biochemical analysis, and to calculate the area *vs* DW ratio.

### Photosynthesis-Irradiance (P-I) response curves and Chlorophyll fluorescence imaging (CFI)

P-I curves (*n* = 4) were performed accordingly to Silva et al.^75^, after the heatwave peak (HW, C) and after heatwave recovery (HW/R, C/R). The setup, installed right next to the mesocosm facility, was composed of five independent chambers, each with a round plastic PVC chamber filled with water from the aquaria and sealed with a petri dish containing an optical O_2_ sensor (Presens Spot PS; Supplementary Fig. S8).Water temperature inside the chambers was kept at 28 °C (HW) and 20 °C (C, HW/R, C/R) by a closed-circuit thermostatic water-bath temperature controller (Julabo HC, Julabo Labortechnik, Seelbach, Germany). A magnetic stirrer ensured water homogenisation inside the chambers. Light energy was provided by five LED lamps, whose irradiance was previously measured with a Li-Cor LI-190 cosine quantum sensor (LI-COR, Lincoln, NE, USA). Combinations of neutral density filters were used to obtain the different light intensities needed. Leaf samples were cleaned from epiphytes; the middle part was cut into 3 segments (≈ 5 cm long) and then placed side by side inside the chambers to ensure their even exposure to light. Leaf segments were incubated inside the chambers under increasing photosynthetically active radiation (PAR), with ten light levels ranging from 0 to 1372 μmol photons m^−2^ s^−1^. Light intensities used for measurements were chosen beforehand to draw an accurate P-I curve shape. O_2_ concentration (μmol L^−1^) was measured in each chamber, firstly after 20–30 min incubation in the dark (dark respiration, DR) and then under each light intensity (net photosynthesis, NP) with a Microx 4 PreSens Optode (Regensburg, Germany). Photosynthetic rates (DR, NP (1) and GP (2); μmol O_2_ gDW^−1^ h^−1^) were calculated as follows:

1 $$ DR, NP = \frac{{\frac{{\left[ {O_{2} } \right]_{f} - \left[ {O_{2} } \right]_{i} }}{T} \times V}}{DW} $$

where $\left[ {O_{2} } \right]_{f}$ = Final O_2_ concentration (μmol L^−1^); $\left[ {O_{2} } \right]_{i}$ = Initial O_2_ concentration (μmol L^−1^); $T$ = Incubation time (h); $V$ = Volume of the chamber (L); $DW$ = Dry weight of the leaf tissue (g)

2 $$ GP = NP + DR $$

O2 saturation levels were periodically checked during the measurements, and incubation time was adjusted to avoid O_2_ supersaturation in the chamber, which can inhibit photosynthesis and involve pH changes^54,76^. GP response to PAR was analysed using the Jassby & Platt model^77,78^ with SigmaPlot for Windows (version 14.0, 2017 Systat Software, Inc.). Maximum photosynthetic rate (P_m;_ µmolO_2_ gDW^−1^ h^−1^) and photosynthetic efficiency (α; µmolO_2_ gDW^−1^ h^−1^/µmol photons m^−2^ s^−1^) were calculated from the Jassby & Platt fit model, and the half-saturation irradiance (I_k;_ µmol photons m^−2^ s^−1^) was calculated according to the following equation:

3 $$ I_{k} = \frac{{P_{m} }}{{\upalpha }} $$

CFI was done right after leaves sampling with an IMAG-K2 Imaging-PAM Fluorometer (M-Series Chlorophyll Fluorescence System, WALZ, Germany). A 0.8 s saturating light pulse (ca. 5000 µmol photons m^−2^ s^−1^) was applied to each sample immediately before taking the image. Φ_PSII_ is widely used to assess the level of plant stress in seagrasses ^54^, as is it highly sensitive to stress. Φ_PSII_ in ambient light conditions was computed from each image by “point measurements”, according to the following equation^79^ :

4 $$ {\Phi }_{PSII} = (F^{\prime}_{m} - F_{s} )/F^{\prime}_{m} = \Delta F/F^{\prime}_{m} $$

where $F^{\prime}_{m}$: Maximum fluorescence of the light-adapted leaf tissue; $F_{s}$: Steady-state fluorescence of the light-adapted leaf tissue.

For each leaf sampled, three images were taken, one per tissue age (*young*, *mature* and *old*). For each tissue age, three replicates of areas of interest (AOI) were selected on the leaf’s image to calculate mean Φ_PSII_.

### Total phenolic content (TPC), Trolox® equivalent antioxidant activity (TEAC), oxygen radical absorbance capacity (ORAC) and malondialdehyde (MDA) quantification

Foliar antioxidant biochemical indicators (Total Phenolic Content, TPC, Trolox® Equivalent Antioxidant Capacity, TEAC, and Oxygen Radical Absorbance Capacity, ORAC) and oxidative damage (Malondialdehyde, MDA) were investigated. Following collection, leaf samples were carefully cleaned from epiphytes, rinsed with distilled water, blotted dry, frozen in liquid nitrogen, and stored at – 80 °C until analysis.

TPC, TEAC and ORAC were quantified according to Costa et al.^35^. 0.15 g of frozen leaf samples were powdered in liquid nitrogen, suspended in 2.5 mL of hydrochloric acid (HCL) 0.1 N, kept overnight in the dark under constant shaking at 4 °C, and then centrifuged (4700 xg, 30 min, 4 °C). The supernatant was used to quantify TPC and for TEAC and ORAC assays.

TPC was quantified using the Folin-Ciocalteu method^80,81^. 42 µL of the phenolic extract was added to 0.4 mL Folin-Ciocalteu reagent 0.25 N and 0.4 mL of NA_2_CO_3_ 7.5%. Absorbance was read at 724 nm (Novaspec Plus, Healthcare Bio-Sciences AB, Uppsala, Sweden). Chlorogenic acid was used as a standard, and TPC was expressed as chlorogenic acid equivalents.

The TEAC assay quantifies the protection capacity against peroxyl radicals (ROO•) through total antioxidant capacity based on a single electron transfer. A cation radical ABTS^•+^ solution was produced by adding 7 mM ABTS to potassium persulfate (2.45 mM final concentration), according to Re et al.^82^. 990 μL of diluted ABTS^•+^ solution (absorbance 0.8 ± 0.02 at 734 nm) was added to 10 μL of extract and read at 734 mm (Novaspec Plus, Healthcare Bio-Sciences AB, Uppsala, Sweden). Results were expressed as Trolox® ﻿(6-Hydroxy-2,5,7,8-tetramethylchromane-2-carboxylic acid) equivalents.

The ORAC assay quantifies the protection capacity against peroxyl radicals through hydrogen atom transfer (HAT). The ORAC analysis was done following Gillespie et al.^83^ and Huang et al.^84^, and using ABAP ﻿[2,2′-azobis (2-methylpropionamidine) dihydrochloride] instead of AAPH [2,2′-azobis(2-amidinopropane) dihydrochloride] as a lipophilic peroxyl radical generator. 150 µL of 8.2 × 10^–5^ mM fluorescein in 75 mM potassium phosphate buffer (pH 7.4) was added to 25 µL of extract, heated to 37 °C and read in a Synergy TM 4 multi-detection microplate reader (485 nm excitation filter, 20 nm bandpass, and 528 nm excitation filter, 20 nm bandpass). The reaction was initiated by adding 25 µL of freshly prepared 153 mM ABAP. The results were expressed as Trolox® equivalents.

MDA is a final secondary product of polyunsaturated fatty acids autooxidation (responsible for cell damage) and enzymatic degradation. Hence, it is considered a valuable indicator of lipid peroxidation under oxidative stress^85^. MDA extraction and quantification was performed as in Hodges et al.^85^. 300 mg of frozen leaf tissue were ground in liquid nitrogen and suspended in 5 mL ethanol 80%. After homogenization, the extracts were centrifuged at 3000xg for 10 min. 1 mL of supernatant was added to 1 mL of 20% trichloroacetic acid (TCA) with 065% thiobarbituric acid (TBA) and 0.015% butylated hydroxytoluene (BHT) solution. Two blank solutions were made without TBA or with ethanol 80% instead of sample extract. After mixing well, all samples and blanks were incubated at 90 °C for 25 min, cooled down in ice for 15 min, and centrifuged at 3000 xg for 10 min. The supernatant absorbances were read at 440, 532 and 600 nm (Novaspec Plus, Healthcare Bio-Sciences AB, Uppsala, Sweden), and MDA equivalents were calculated as in Hodges et al.^85^.

### Leaf area vs dry weight ratio

Leaves’ area *vs* dry weight (DW) ratio was calculated. Leaf segments were photographed for later measurement of their surface area (m^2^) with the ImageJ software^86^ and each sample’s DW was measured after drying at 60 °C for at least 48 h.

### Statistical analyses

All statistical analyses were performed using R Studio software^87^. Beforehand, data were tested for normality (Shapiro–Wilk’s test) and homogeneity of variances (Levene’s test). Differences between treatments (HW *vs* C) at both sampling times (“*heatwave*” and “*heatwave recovery*”) were tested using one-way analysis of variance (ANOVA). Whenever the hypothesis of homogeneity of variance was rejected, a Welch ANOVA test was performed, followed by a Games-Howell post-hoc test. To investigate the coupled effects of tissue age and treatment on Φ_PSII_, a two-way ANOVA was performed. In case of the absence of significant interaction between the two factors, two one-way ANOVAs were performed to search for any significant difference in Φ_PSII_ between leaf parts and between treatments, independently. If significance was detected, a Tukey-HSD test was performed for pairwise comparison of the factors “leaf part” and “treatment”. For all tests, a significance level of α = 0.05 was used. Data points that deviated from the upper and lower quartiles more than 1.5-fold the interquartile range were considered outliers and were not included in the analysis^88^.

## Supplementary Information

Supplementary Information.

## Data Availability

The datasets generated and/or analysed during the current study are not publicly available due to confidentiality reasons, but are available from the corresponding author on reasonable request.
