# Supplementary material for: Physiological and morphological effects of a marine heatwave on the seagrass Cymodocea nodosa
Source: Sci Rep. 2022 May 13;12:7950. doi: 10.1038/s41598-022-12102-x (PMC9106744; doi:10.1038/s41598-022-12102-x)
Supplement: Supplementary file 1 — Supplementary Information. [file 41598_2022_12102_MOESM1_ESM.docx]

**Supplementary** **material**

**Physiological and morphological effects of a marine heatwave on the seagrass *Cymodocea nodosa***

Alizé Deguette^1^, Isabel Barrote^1*^, João Silva^1*^◊

**Supplementary Figure S1**: Satellite view of the Ria Formosa lagoon between Faro and Olhão, on the South coast of Portugal. *C. nodosa* sampling site is marked in red (Google satellite, WGS84).


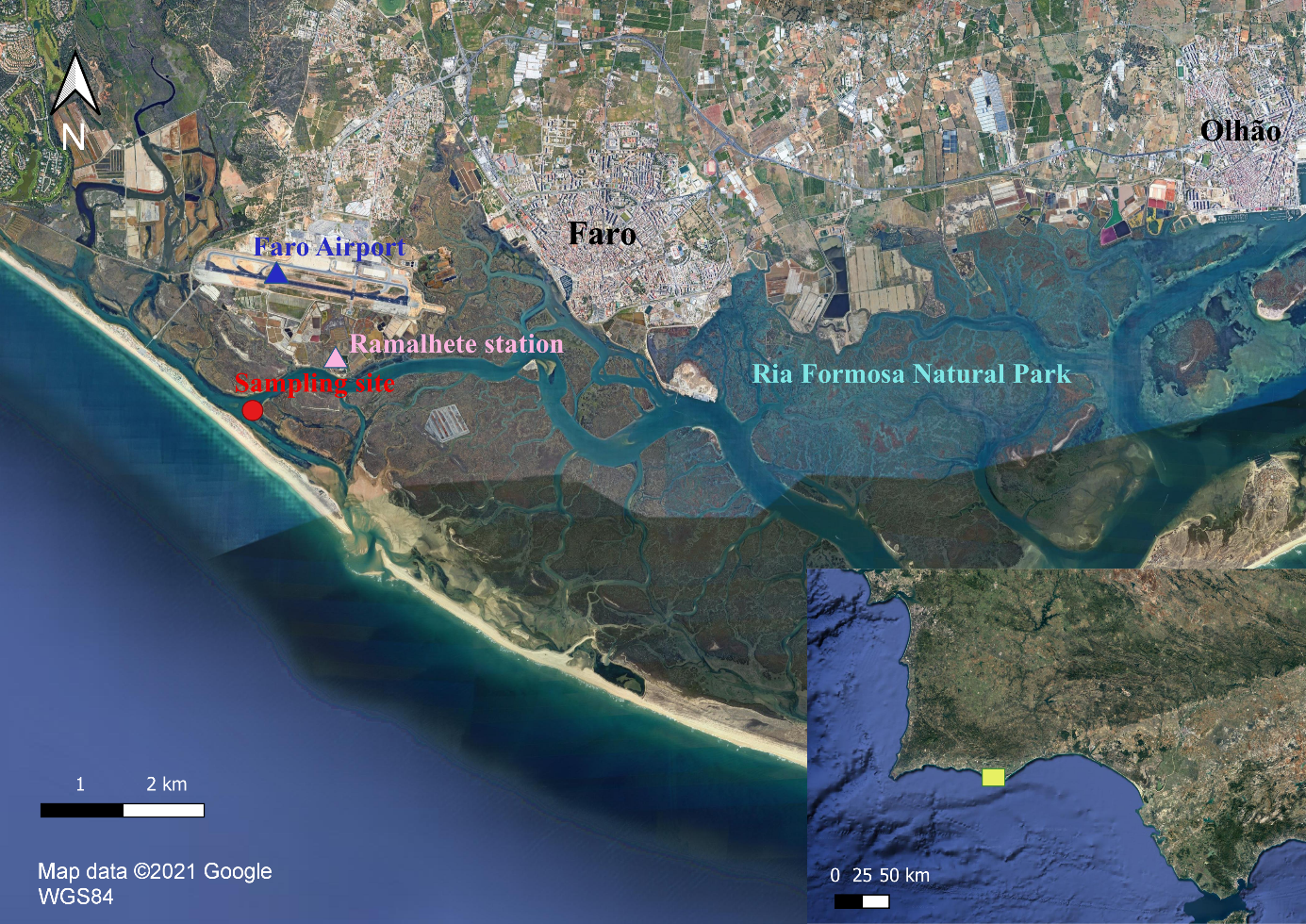


**Supplementary Figure S2**: Mesocosm facility at Ramalhete Field Station (CCMAR). Ten 65-L plastic tanks (5 replicates per treatment, *n=*5) were filled with 15 cm of sand and supplied with water pumped directly from Ria Formosa, running through an open circuit.


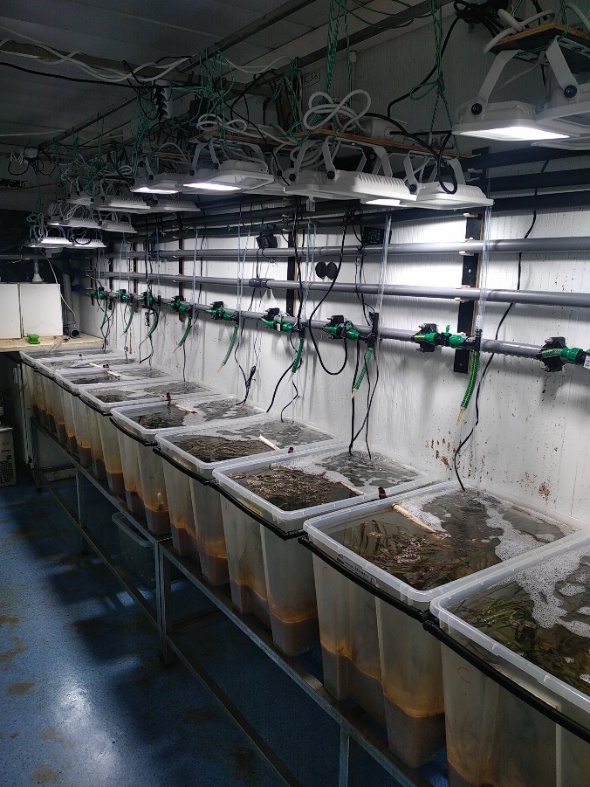


**Supplementary Figure S3**: Time series of oceanic water SST adjacent to Ria Formosa, from 10-06-2017 to 17-06-2019 (black line). Blue scattered line: climatology; red dotted line: threshold; orange-coloured: marine heatwave; red-coloured: heatwave peak. Red marks on the x-axis indicate MHWs events. (Adapted from the Marine Heatwave Tracker app; Schlegel, 2020)


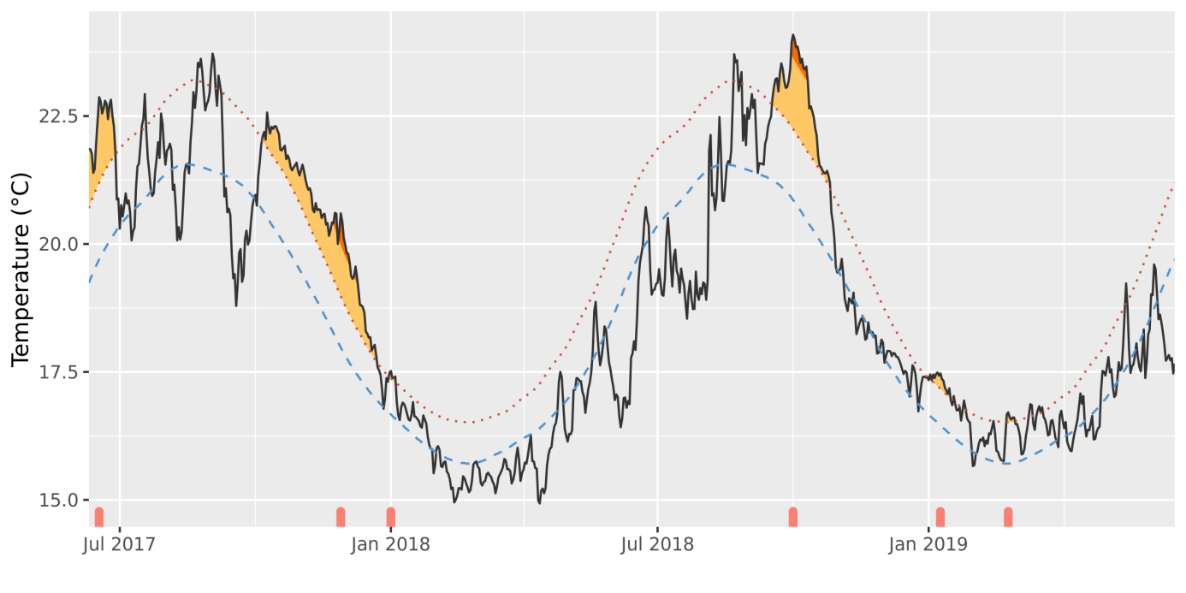


**Supplementary Figure S4**: Linear regression plot assessing the correlation between the water temperature inside and outside Ria Formosa (SST, data recorded by an oceanographic buoy of the Instituto Hidrográfico) in 2018, and corresponding correlation equation. Data frequency is every five days.


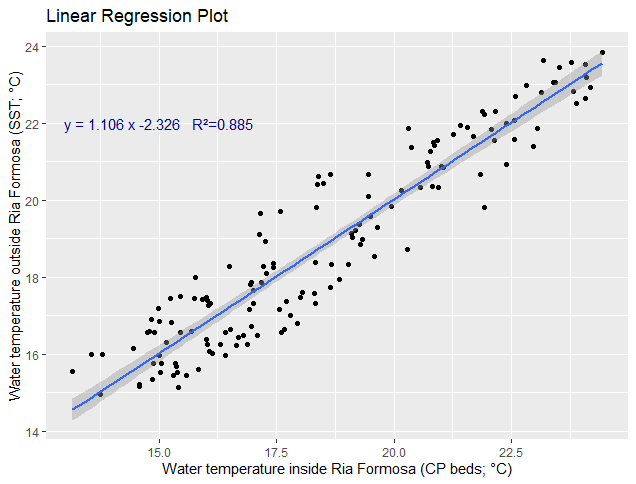


**Supplementary Figure S5**: Oceanic SST climatology (from NOAA OISST) and threshold, and extrapolated Ria Formosa’s (RF) SST climatology and threshold for the year 2018 (left) and for April 1st to June 30th (right), according to the correlation equation y= 1.106x -2.32

**Supplementary Figure S6**: Occurrence and intensity of MHWs in the area adjacent to Ria Formosa from 1982 to the present day. Blue arrows show MHWs occurring in the April-June period. (Adapted from the Marine Heatwave Tracker app; Schlegel, 2020)

**Supplementary Figure S7**: MHWs classification by Hobday et al. (2018) applied to the extrapolated Ria Fomosa’s temperature data. Ria Formosa’s climatology (black), 90^th^ percentile (grey), multiples of the 90^th^ difference (2x, 3x and 4x, yellow, orange and red, respectively) from the mean climatology value. Coloured lines represent the minimum temperature of the corresponding interval (e.g., the orange line is the minimum temperature for a MHW of Severe intensity (category III). A MHW of Severe intensity has its peak temperature between the orange and the red line). A MHW of Extreme intensity (category IV) is defined by a peak temperature above the red line.


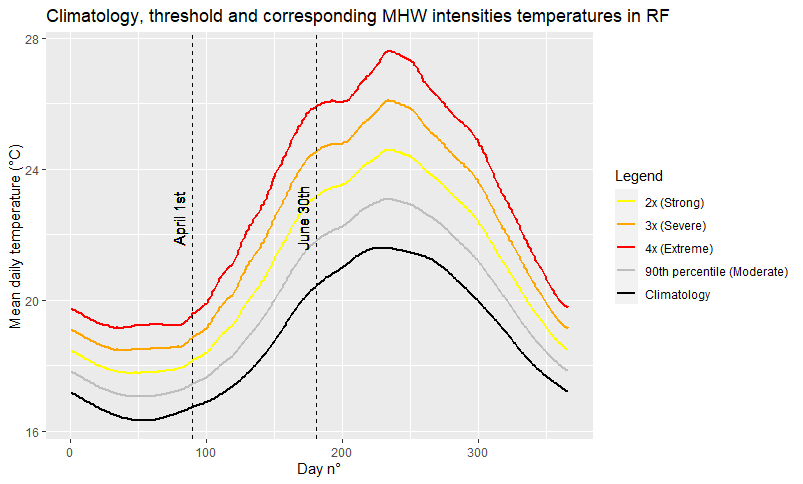


**Supplementary Figure S8**: Setup used to do the P-I curves. a) five independent chambers with respective light source; b) top-view of one chamber, showing the PVC incubation chamber, magnetic stirring, and squared frame to install the density filters; c) zoom of the top of one incubation chamber, with leaf samples placed horizontally and apparent O_2_ sensor (green dot).
